# Tuberculose pariétale compliquée d’embolie pulmonaire

**DOI:** 10.11604/pamj.2017.27.107.10503

**Published:** 2017-06-12

**Authors:** Regis Gothard Bopaka, Presley Lee Esthel Bemba, Hind Janah, Franck Hardain Okemba Okombi, Hasna Jabri, Wiam El Khattabi, Hicham Afif

**Affiliations:** 1 Service de Pneumophtisiologie, CHU de Brazzaville, Congo; 2 Service des Maladies Respiratoires, Hôpital 20 Août 1953, CHU Ibn Rochd, Casablanca, Maroc

**Keywords:** Tuberculose pariétale, embolie pulmonaire, bilan étiologique, Parietal tuberculosis, pulmonary embolism, etiologic assessment

## Abstract

La tuberculose est une maladie infectieuse qui reste fréquente dans les pays en développement. La localisation peut être pulmonaire ou extra-pulmonaire. Cette forme extra-pulmonaire pose un grand problème diagnostique. Nous rapportons le cas d'un patient présentant une embolie pulmonaire révélant une tuberculose pariétale. A travers cette observation, nous soulignons la nécessité de rechercher l'étiologie devant une embolie pulmonaire.

## Abstract (en)

Tuberculosis is a frequent infectious disease in developing countries. It can affect the lung or spread to other parts of the body. Extra-pulmonary tuberculosis poses a major diagnostic problem. We report the case of a patient with pulmonary embolism revealing parietal tuberculosis. This study emphasizes the importance of etiologic assessment in patients with pulmonary embolism.

## Introduction

La tuberculose pulmonaire et extra-pulmonaire est une maladie infectieuse encore fréquente dans les pays en développement. La localisation extra-pulmonaire tend à se rapprocher à l'atteinte pulmonaire d'après les données épidémiologiques actuelles [1]. Cette forme extra-pulmonaire pose un grand problème diagnostique [2]. Nous rapportons le cas d'un patient présentant une tuberculose pariétale révélée par une complication d'embolie pulmonaire. A travers cette observation, nous soulignons la nécessité de rechercher l'étiologie devant une embolie pulmonaire.

## Patient et observation

**Figure 1:**
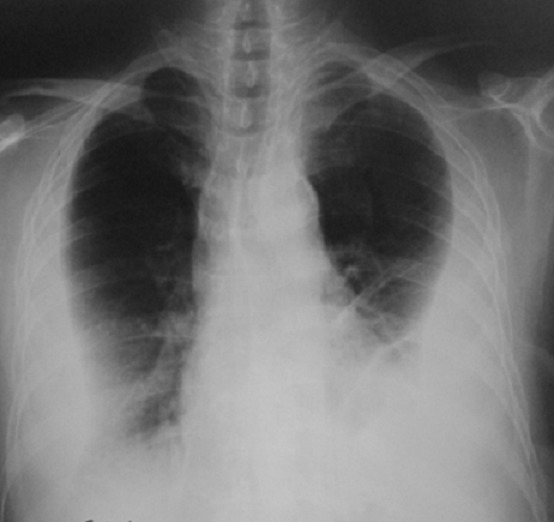
Radiographie du thorax: opacité bilatérale de type pleural

Monsieur B. A âgé de 54 ans était un ex-tabagique sevré il y a 5 ans et n'a pas d'antécédent pathologique particulier. Il rapportait depuis 2 mois une toux sèche, une douleur thoracique diffuse et atypique et une dyspnée d'effort évoluant dans un contexte de fléchissement de l'état général ayant imposé un alitement prolongé. L'examen clinique à l'admission trouvait un patient en assez bon état général, apyrétique à 37°C, et polypnéique à 28 cycles/min. Il était normo-tendu et tachycarde à 110 batt/min. L'examen pleuro-pulmonaire a objectivé un syndrome d'épanchement liquidien basithoracique bilatéral. Le reste de l'examen clinique était sans particularité. La radiographie thoracique avait objectivé une opacité bilatérale de type pleural (Figure 1), sans lyse osseuse ni une lésion des parties molles. La ponction pleurale bilatérale a mis en évidence un liquide jaune citrin, exsudatif et lymphocytaire à 90%. La recherche de bacille de Koch (BK) à l'examen direct et culture dans le liquide pleural était négative. Les ponctions-biopsies pleurales n'ont montré qu'une inflammation chronique non spécifique. La culture des fragments de biopsies pleurales à la recherche de BK était négative. Les diagnostics discutés étaient une pleurésie bilatérale d'origine maligne (métastatique ou lymphomateuse), tuberculeuse, dans le cadre d'une maladie de système (lupus érythémateux systémique ou polyarthrite rhumatoïde). Le bilan biologique a montré une numération sanguine normale, la vitesse de sédimentation était accélérée à 112 mm à la 1ère heure. L'intradermo-réaction à la tuberculine était négative. Les sérologies virales (Virus de l'Immunodéficience Humaine, hépatites B et C) et l'électrophorèse des protéines étaient normales. L'échographie abdominale était sans particularités et le dosage de l'antigène prostatique spécifique (PSA) était négatif. D'autres ponction-biopsies pleurales en regard de l'épaississement pleural étaient négatives.

**Figure 2:**
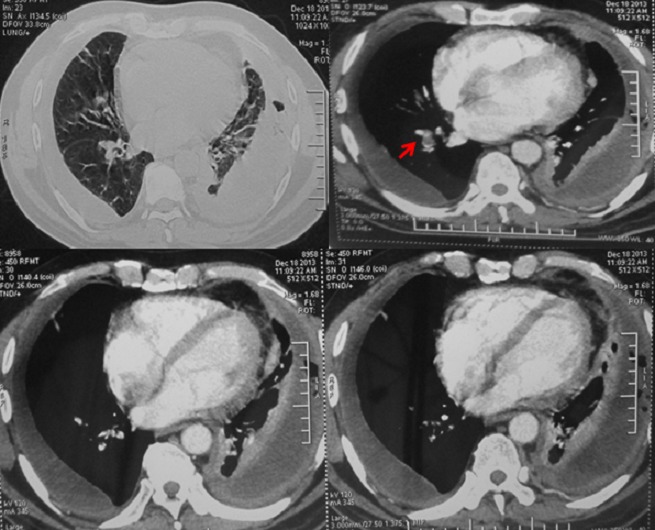
Angioscanner thoracique: embolie pulmonaire du lobe inférieur droit associée à une pleurésie bilatérale et un épaississement pleural gauche

**Figure 3:**
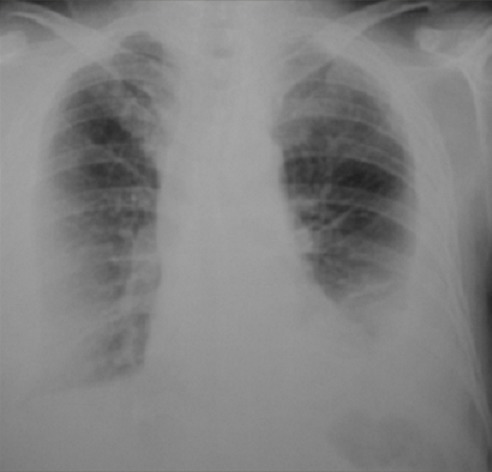
Radiographie du thorax: opacité bilatérale de type pleural en regression

**Figure 4:**
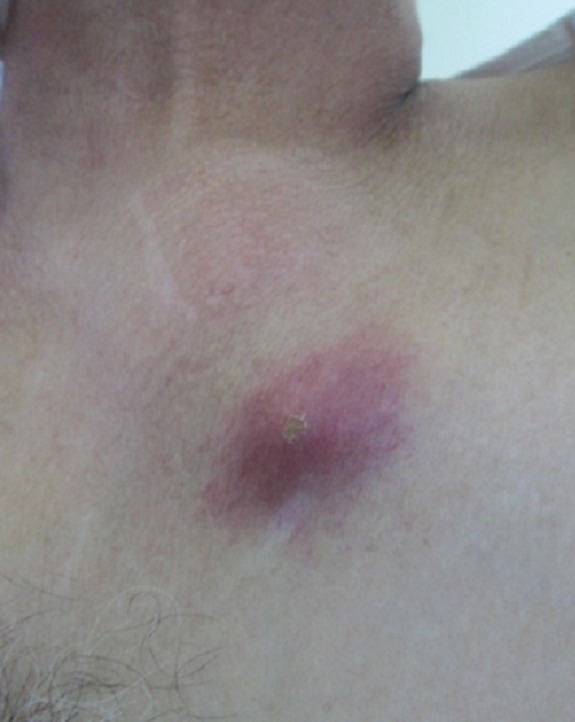
Examen clinique: tuméfaction de la face antérieure du thorax avec des signes inflammatoires en regard

**Figure 5:**
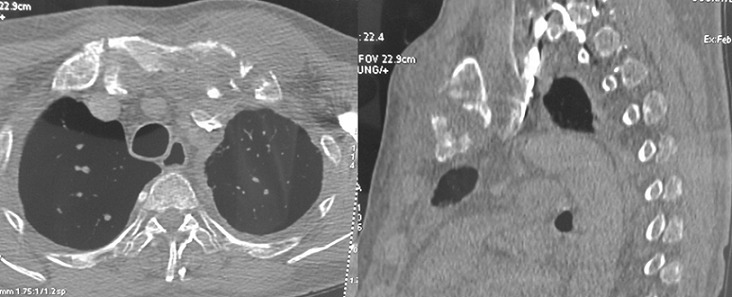
Tomodensitométrie cervico-thoracique: masse pariétale avec lyse osseuse du manubrium sternal

**Figure 6:**
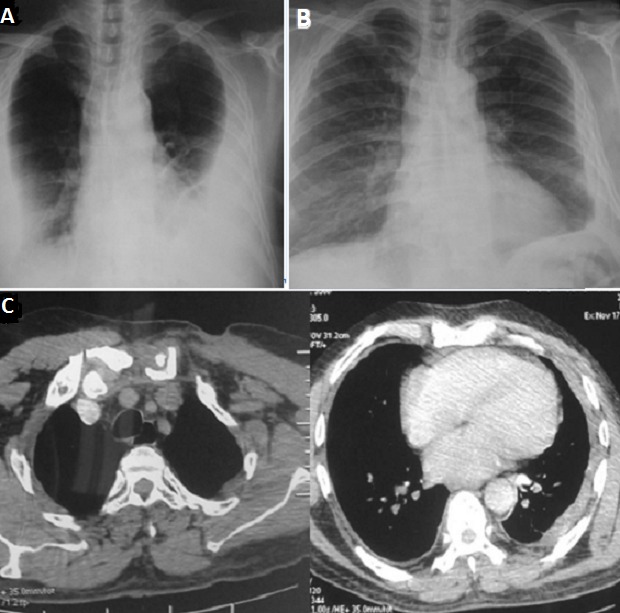
(A) radiographie du thorax à l’admission: opacité bilatérale de type pleural; (B) radiographie du thorax à la fin du traitement: régression de l’opacité bilatérale; (C) tomodensitométrie thoracique: régression de la pleurésie et de la masse pariétale

Malgré l'évolution progressive, une embolie pulmonaire pouvant être associée ou non aux diagnostics précédents était évoquée devant l'aggravation de la polypnée à 30 cycles/min et l'alitement prolongé. La probabilité clinique était intermédiaire selon le score de Wells (fréquence cardiaque>100, immobilisation récente). Les d-dimères étaient à 7500 ng/ml (valeur normale < 500). L'angioscanner thoracique a objectivé une embolie pulmonaire du lobe inferieur droit (Figure 2) associée à une pleurésie bilatérale et un épaississement pleural gauche inhabituel dans l'embolie laissant suspecter une autre pathologie sous-jacente notamment tuberculeuse ou maligne. Le patient a été mis sous traitement anticoagulant. L'évolution après 1 mois a été marquée par la régression de la dyspnée et de l'épanchement pleural (Figure 3). Après 2 mois de traitement anticoagulant, le patient a présenté avec une tuméfaction latéro-sternale gauche avec à la tomodensitométrie thoracique une masse pariétale avec lyse osseuse (Figure 4, Figure 5). La ponction-biopsie transpariétale a ramené du matériel inflammatoire sans spécificité. La biopsie chirurgicale a montré une infiltration granulomateuse tuberculoïde largement nécrosante compatible avec une tuberculose caséo-folliculaire sans signes de malignité. Au final, il s'agissait d'une tuberculose pariétale (abcès froid) compliquée d'une embolie pulmonaire comme mode révélateur sur un terrain immunocompétent. Le traitement antibacillaire a été démarré avec surveillance du bilan d'hémostase vu les interactions médicamenteuses possibles notamment entre anti-vitamines K et rifampicine. L'évolution a été favorable à 7 mois du traitement antituberculeux avec régression de la pleurésie et de la masse pariétale (Figure 6).

## Discussion

La tuberculose est un véritable problème de santé au Maroc. L'incidence toutes formes de tuberculose est de 82 cas pour 100 000 habitants en 2014. La tuberculose extra-pulmonaire représente 46,2% des cas [1]. L'embolie pulmonaire est une complication de la tuberculose pulmonaire qui a reçu peu d'attention dans la littérature [3]. C'est une complication rare mais qui peut mettre en jeu le pronostic vital [4]. L'association entre l'inflammation due à la tuberculose et un état d'hypercoagulabilité a été décrite [4]. La pleurésie peut être le mode révélateur de la tuberculose et d'embolie pulmonaire. Dans les deux affections, la pleurésie peut être unilatérale ou plus rarement bilatérale. Dans cette observation, l'évolution chronique et l'épaississement pariétal gauche rendaient la probabilité d'une tuberculose pleurale gauche plus élevée. L'atteinte pleurale du côté droit est plutôt rattachée à l'embolie vu les données du scanner. Une embolie pulmonaire bilatérale ne peut cependant être écartée. La biopsie chirurgicale de la masse pariétale a été d'un grand apport pour le diagnostic étiologique. L'évolution sous traitement spécifique des deux affections permet en général d'obtenir la guérison. Il faut cependant faire attention aux interactions vu que la rifampicine est un fort inducteur enzymatique [5, 6].

## Conclusion

A travers cette observation, nous soulignons la difficulté de diagnostic de la tuberculose extra pulmonaire et la nécessité de bilan étiologique d'une embolie pulmonaire. Les cliniciens doivent être conscients du risque de développer une maladie thromboembolique chez les patients traités pour tuberculose et qu'une embolie pulmonaire peut masquer une tuberculose notamment dans les pays endémiques.

## Conflits d’intérêts

Les auteurs ne déclarent aucun conflit d'intérêts.

## References

[1] Plan National d'accélération de la réduction de l'incidence de la tuberculose 2013-2016 au Maroc..

[2] Cherif J, Mjid M, Ladhar A, Toujani S, Mokadem S, Louzir B (2014). Délai diagnostique de la tuberculose pulmonaire et pleurale. Rev Pneumol Clin..

[3] Ekukwe NC, Bain LE, Jingi AM, Sylvia L, Mintom P, Menanga A (2014). Bilateral pulmonary embolism in a patient with pulmonary tuberculosis: a rare association in Yaoundé Cameroon. Pan Afr Med J..

[4] Gonclaves IM, Alves DC, Carvalho A, Do Brito MC, Calvario F, Duarte R (2009). Tuberculosis and venous thromboembolism. Case J..

[5] Tisserand G, Gil H, Méaux-Rault N, Magy-Bertrand N (2014). Particularités cliniques de l'embolie pulmonaire chez la personne âgée: étude comparative de 64 patients. Rev Med Intern..

[6] Aristoff PA, Garcia GA, Kirchhoff PD, Hollis Showalter HD (2010). Rifamycine-obstacles and opportunities. Tuberculosis (Edinb)..

